# The flea on the Magnetic Elephant

**DOI:** 10.1007/s11005-026-02055-x

**Published:** 2026-02-27

**Authors:** Pavel Exner, Léo Morin

**Affiliations:** 1 Doppler Institute for Mathematical Physics and Applied Mathematics, Czech Technical University, Břehová 7, 11519 Prague, Czechia; 2 Department of Theoretical Physics, NPI, 25068 Řež near Prague, Czechia; 3 Department of Mathematics, University of Copenhagen, Universitetsparken 5, 2100 Copenhagen Ø, Denmark

**Keywords:** Spectral theory, Magnetic laplacian, Tunneling, Semiclassical limit, 35P15, 35Q40, 81Q10, 81Q20

## Abstract

We investigate a two-dimensional magnetic Laplacian with two radially symmetric magnetic wells. Its spectral properties are determined by the tunneling between them. If the tunneling is weak and the wells are mirror symmetric, the two lowest eigenfunctions are localized in both wells being distributed roughly equally. In this note, we show that an exponentially small symmetry violation can in this situation have a dramatic effect, making each of the eigenfunctions localized dominantly in one well only. This is reminiscent of the ‘flea on the elephant’ effect for Schrödinger operators; our result shows that it has a purely magnetic counterpart.

## Introduction

One of the remarkable effects in quantum mechanics concerns the situation when a tiny violation of symmetry of a double-well system may cause a dramatic change in the associated bound state wave functions. B. Simon [27] gave it the name ‘flea on the elephant’ when he extended an earlier work done in [10, 22] to a multidimensional setting; in his words, the flea does not change the shape of the elephant but it can irritate the elephant enough so that it shifts its weight.

The crucial element in understanding of this effect was an analysis of tunneling between symmetric wells, specifically the construction of an interaction matrix which effectively describes the bottom of the spectrum and the associated eigenfunctions (see [19, 27]). Tunneling in magnetic systems turned out to be more complicated, and for a long time only partial results were known [20]. The first purely magnetic tunneling formula was proved in [2], then followed by [1, 8]. The common feature in these works is a multiscale behavior of the eigenfunctions, which makes tunneling effectively occur along a curve. However, in the case of two-dimensional wells, there is no such multiscale behavior, thus making the analysis mathematically challenging.

The first significant improvement in many years was [3], where the case of symmetric double electric wells with homogeneous magnetic field was analyzed. Under radiality assumptions on the wells, [3] provides first lower bounds on tunneling for arbitrary magnetic field strength. This motivated other works to improve the bounds on the spectral gap, which measures the intensity of tunneling [15, 16], finally leading to a sharp tunneling formula in this case [23]. These works highlight the influence of fast oscillations of the eigenfunctions on tunneling, a phenomenon which does not occur without magnetic field. The non-radial situation is still widely open, and the recent contribution [4] illustrates the strong effect of magnetic fields, by giving examples when tunneling can be killed, even if the wells are symmetric. However, such examples should be exceptional, as a positive lower bound on average was proven in [5].

In this article, we focus on the purely magnetic analogue of the above. An analysis of purely magnetic and symmetric double wells was carried out in [9], under radiality assumptions on the wells. It should be noted that this two-dimensional tunneling between magnetic wells is a long-standing open problem that was raised in the 90’s, see [18]. Recently in [6], the first example of tunneling formula between non-radial purely magnetic wells was obtained. The proof, relying on semiclassical phase-space analysis, enlightens the role of the classical center guide motion, which is at the origin of tunneling.

The aim of the present note is to show that the ‘flea on the elephant’ effect has a purely magnetic counterpart which shares with the original the mechanism based on tunneling between two sufficiently separated wells. Imposing the radiality assumptions on the wells, we adapt the techniques from [9] and incorporate *perturbations* of a purely magnetic symmetric double well. We show that tiny perturbations can again have a dramatic effect on the eigenfunctions.

Speaking about magnetic wells, we do not have in mind any particular physical system, but there are many situations where such a spectral problem can arise. Some are associated with local manipulations of a material’s magnetic properties [21] which are the essence of most of the currently used memory devices. But there are also other ways, for instance, placing a superconducting mask on the top of a semiconductor thin film exposed to a magnetic field, one screens the field out (almost completely) in that region in view of the Meissner effect [26] creating exactly a well of the type considered here. We refer to [7] for further discussions on the link between the Ginzburg–Landau theory of superconductivity and the spectrum of magnetic Laplacians.

In the next section, we formulate the problem and state our main result, Theorem 2.3, reducing the semiclassical expansion at the bottom of the spectrum to a $2\times 2$ matrix problem. This theorem generalizes the results from [9], since the two wells do not need to be symmetric. In other words, each single-well problem needs only to have close enough ground state energies that are not necessarily equal. Corollary 2.7 gives a precise description of the flea on the elephant effect that is the behavior of the eigenfunctions when a perfectly symmetric double-well system is perturbed. Section 3 is then devoted to the proof of the main result, Theorem 2.3.

## Perturbations of symmetric magnetic wells

### The problem setting and preliminaries

As indicated, we will be concerned with purely magnetic double wells; our system is two-dimensional and exposed to magnetic field perpendicular to the plane. Specifically, we consider a pair of *radial* magnetic wells centered at $x_\ell = \big (-\frac{L}{2},0\big )$ and $x_r=\big (\frac{L}{2},0\big )$; the respective left and right fields are given by $\mathscr {C}^\infty$-smooth functions $B_\ell$ and $B_r$, such that

- $B_\star >0$ is a radial function of $x-x_\star$,
- $B_\star$ has its unique minimum at $x_\star$ which is non-degenerate,
- $$B_\star (x) = b_1$$ holds for $$|x-x_\star |\geqslant a$$,

where $\star \in \lbrace \ell , r \rbrace$, and $a \in \big (0,\frac{L}{2}\big )$ and $b_1 >0$ are some constants. The double-well magnetic field *B* is then defined as superposition of the two single wells,

2.1 $$\begin{aligned} B(x) = {\left\{ \begin{array}{ll} B_\ell (x) & {\text {if}} \quad x_1 \le 0,\\ B_r(x) & {\text {if}} \quad x_1 \ge 0. \end{array}\right. } \end{aligned}$$

With each of the fields $B_\ell$ and $B_r$, we associated a vector potential satisfying $\nabla \times A_\star = B_\star$; we choose for them the circular gauge,

$$\begin{aligned} A_\star (x) = \int _{[x_\star ,x]} B_\star (y) (y-x_\star )^\perp \textrm{d}y, \quad y^\perp = (-y_2,y_1), \quad \star \in \lbrace \ell , r \rbrace . \end{aligned}$$

Let us first recall some known facts about single-well system properties. The operators describing the individual wells are then given by

$$\begin{aligned} \mathscr {L}_\star = (ih\nabla + A_\star )^2, \quad \star \in \lbrace \ell ,r \rbrace . \end{aligned}$$

Since each of the magnetic fields $B_\star$ has by assumption a unique and non-degenerate minimum, it is well known [11, 17, 25] that the bottom of the spectrum of $\mathscr {L}_\star$ is discrete and asymptotic expansion for the low eigenvalues can be obtained explicitly. In particular, according to [11, Theorem 1.2] we have

#### Theorem 2.1

For $\star \in \lbrace \ell , r \rbrace$, the ground state of $\mathscr {L}_\star$ is a radial function of $x-x_\star$. Moreover, the eigenvalues at the bottom of the spectrum of $\mathscr {L}_\star$ satisfy

$$\begin{aligned} \mu _{\star ,j}(h) =h \min B_\star + h^2 \big ( 2j d_\star + d'_\star \big ) + \mathscr {O}(h^3), \end{aligned}$$

with

$$\begin{aligned} d_\star = \frac{\sqrt{\det H_\star }}{\min B_\star }, \qquad d'_\star = \frac{\big ( {\textrm{tr}} H_\star ^{\frac{1}{2}}\big )^2}{2\min B_\star }, \qquad H_\star = \frac{1}{2}\, \mathrm{{Hess}} B_\star (x_\star ). \end{aligned}$$

In the following, we will use for simplicity the symbol $\mu _\star := \mu _{\star ,1}$ for the ground state energy of $\mathscr {L}_\star$, and by $\Psi _\star$ we denote the corresponding ground state eigenfunction. Since it is a radial function, the eigenvalue equation becomes the 2D radial Schrödinger equation,

$$\begin{aligned} -\frac{1}{r} \partial _r r \partial _r \Psi _\star + \frac{1}{r^2} \Big ( \frac{1}{2\pi } \int _{D(x_\star ,r)} B_\star \mathrm d x \Big )^2 \Psi _\star = \mu _\star \Psi _\star , \end{aligned}$$

in the coordinate $r=|x-x_\star |$. In view of this observation, we can construct WKB approximations of $\Psi _\star$ in the spirit of [14, 19]. Note that such expansions can also be obtained in a magnetic non-radial setting [11, 12], however, these are not known to be exponentially good approximations.

#### Proposition 2.2

For $\star \in \lbrace \ell , r \rbrace$, we put

$$\begin{aligned} d_\star (s):=\int _{0}^{s}\frac{1}{2 \pi v^2} \left( \int _{D(x_\star ,v)} B_\star \textrm{d}x\right) v \textrm{d}v\,. \end{aligned}$$

There exists a sequence of smooth functions $(a_{j})_{j\geqslant 0}$ on $\mathbb {R}^2$, radial with respect to $x-x_\star$, with $a_{0}>0$ such that the following holds: for any given $p\in \mathbb {N}$, setting $a_{h,\star }(x)=\sum _{j=0}^p h^j a_j(x)$, and considering

$$\begin{aligned} \Psi ^{\textrm{WKB}}_\star (x)=h^{-\frac{1}{2}}a_{h,\star }(x)e^{-d_\star (|x-x_\star |)/h}\,, \end{aligned}$$

we have

$$\begin{aligned} e^{d_\star (|x-x_\star |)/h}\left( \mathscr {L}_{\star }-\mu _\star \right) \Psi _\star ^{\textrm{WKB}}=\mathscr {O}(h^{p+1})\,. \end{aligned}$$

Moreover, for all $\epsilon \in (0,1)$, there exist positive $C, h_0>0$ such that, for $h\in (0,h_0)$,

2.2 $$\begin{aligned} \Vert (-ih\nabla -{A}_\star )(e^{(1-\epsilon )d_\star / h}\Psi _\star )\Vert \leqslant Ch\Vert \Psi _\star \Vert \end{aligned}$$

holds for all $h\in (0,h_0)$, and we also have the approximation

$$\begin{aligned} e^{d_\star (|x-x_\star |)/h}(\Psi _{\star }(x)-\Psi _\star ^{\textrm{WKB}}(x))=\mathscr {O}(h^{p+1})\,. \end{aligned}$$

In particular, the inequality (2.2) provides us with exponential decay estimates on the eigenfunction $\Psi _\star$.

### Magnetic double wells

Let us now turn to our proper topic. For the double-well magnetic field (2.1), we choose a vector potential *A* such that $\nabla \times A = B$ and the system dynamics is governed by the self-adjoint realization of

$$\begin{aligned} \mathscr {L}=(ih\nabla +A)^2 \;\; \text { on } \; \mathbb R^2\,; \end{aligned}$$

in contrast with the previous subsection, we do not need to fix a particular gauge.

The central object here is the subspace associated with the spectral projection $\Pi = \textbf{1}_{(-\infty ,\Lambda ]}\big ( \mathscr {L} \big )$, where $\Lambda := \min \big ( \frac{\mu _{\ell }+\mu _{\ell ,2}}{2}, \frac{\mu _r + \mu _{r,2}}{2} \big )$. If the two ground state energies are close to each other, one can construct a basis of $\mathrm{{Ran}} \, \Pi$ using the corresponding left and right well eigenfunctions, $\Psi _\ell$ and $\Psi _r$.

#### Theorem 2.3

Assume that $\mu _\ell - \mu _r = o(h^2)$ and $L> (2+\sqrt{6})a$. Then there exists an orthonormal basis $(e_\ell ,e_r)$ of $\mathrm{{Ran}} \, \Pi$ such that

$$\begin{aligned} e_\ell = \Psi _\ell + \mathscr {O}(h^\infty ), \quad e_r = \Psi _r + \mathscr {O}(h^\infty ), \end{aligned}$$

and the matrix representing $\mathscr {L}$ in this basis is

$$ M = \begin{pmatrix} \mu _\ell &  w \\ \overline{w} &  \mu _r \end{pmatrix} + o(w) $$

with $w= c_0 h^{\nu } e^{-\frac{S}{h}} (1+o(1))$ as $h\rightarrow 0$ for some constants $c_0 \ne 0$ and $\nu \in \mathbb R$. Here

2.3 $$\begin{aligned} S= d_\ell \left( \textstyle {\frac{L}{2}}\right) + d_r \left( \textstyle {\frac{L}{2}} \right) + I, \end{aligned}$$

where the $d_\star$ are defined in Proposition 2.2, *I* is a positive number given by

$$ I= \frac{M}{2} - \frac{b_1L^2}{8} + \frac{b_1L}{2} \sqrt{\frac{L^2}{4}-\frac{M}{b_1}} - M \ln \left( 1 + \sqrt{ 1 - \frac{4\,M}{b_1 L^2}}\right) , $$

and $$M = \frac{1}{2\pi } \int _{\mathbb {R}^2} (2 b_1 - B_\ell - B_r)\,\textrm{d}x$$.

This theorem generalizes the result from [9], by removing the symmetry assumption $B_\ell (x) = B_r(-x)$. We allow for more general pairs of wells, as soon as they have close enough ground state energies $\mu _\ell$ and $\mu _r$. In particular, we can use this result to analyze perturbations of symmetric double wells.

We postpone the proof to Sect. 3 and focus first on the consequences of the theorem. It is easy to diagonalize the $2\times 2$ matrix *M*; as a corollary, we deduce asymptotic behavior of the eigenfunctions of $\mathscr {L}$. The instability of these eigenfunctions we are interested in is then a consequence of the instability of the eigenvectors of *M*.

#### Corollary 2.4

Assume $$\mu _\ell - \mu _r = o(h^2)$$ and $$L>(2+\sqrt{6})a$$.

1. If $|\mu _\ell - \mu _r| \ll w$, then the normalized eigenfunctions $\Psi _1$ and $\Psi _2$ of $\mathscr {L}$ are localized in both wells roughly equally,
  $$\begin{aligned} \int _{x_1 >0} |\Psi _j(x)|^2 \textrm{d}x = \frac{1}{2} + o(1),\quad j=1,2, \end{aligned}$$
  as $$h \rightarrow 0$$.
2. If $|\mu _\ell - \mu _r| \gg w$ with $\mu _\ell < \mu _r$, then the normalized ground state $\Psi _1$ is localized in the left well, and the first excited state $\Psi _2$ in the right well,
  $$\begin{aligned} \int _{x_1>0} |\Psi _1(x) |^2 \textrm{d}x = o(1), \quad \int _{x_1>0} |\Psi _2(x)|^2 \textrm{d}x = 1+o(1), \end{aligned}$$
  as $$h \rightarrow 0$$.

#### Remark 2.5

By symmetry, when $|\mu _\ell - \mu _r | \gg w$ with $\mu _\ell > \mu _r$, the ground state will be localized in the right well instead. The ground state is localized in the deeper well with the smaller ground state energy, and the first excited state is correspondingly localized in the other one.

#### Proof

The matrix *M* introduced in Theorem 2.3 has the eigenvalues

$$\begin{aligned} \lambda _\pm = \frac{\mu _\ell + \mu _r}{2} \pm \frac{1}{2} \sqrt{(\mu _\ell - \mu _r)^2 + 4 |w|^2} + o(w) \end{aligned}$$

with the positive spectral gap $\lambda _+ - \lambda _- = \sqrt{(\mu _\ell - \mu _r)^2 + 4 |w|^2} + o(|w|) >0$. The corresponding eigenvectors satisfy

$$\begin{aligned} v_\pm = \left( \frac{\mu _\ell - \mu _r}{2|w|} \mp \frac{\sqrt{(\mu _\ell - \mu _r)^2 + 4 |w|^2}}{2 |w|}, 1 \right) ^\texttt {T} + o(\Vert v_\pm \Vert ). \end{aligned}$$

Consequently, in case (1) we get

$$ \lambda _{\pm } = \frac{\mu _\ell + \mu _r}{2} \pm |w| + o(|w|), $$

and the normalized eigenvectors are

$$\begin{aligned} \frac{v_\pm }{\Vert v_\pm \Vert } = \frac{1}{\sqrt{2}} {\mp 1 \atopwithdelims ()1} + o(1). \end{aligned}$$

It means that the first two eigenfunctions of $\mathscr {L}$ are of the form

$$\begin{aligned} \Psi _\pm = \frac{1}{\sqrt{2}}( e_\ell \mp e_r ) + o(1) = \frac{1}{\sqrt{2}}( \Psi _\ell \mp \Psi _r ) + o(1), \end{aligned}$$

and the result follows since $\Psi _\ell$ and $\Psi _r$ are exponentially localized near $x=x_\ell$ and $x=x_r$, respectively.

In contrast, case (2) offers a different picture, the eigenvalues and eigenvectors being

$$\begin{aligned} \lambda _- = \mu _\ell + \mathscr {O} \Big ( \frac{|w|^2}{|\mu _\ell - \mu _r|} \Big ), \quad \lambda _+ = \mu _r + \mathscr {O} \Big ( \frac{|w|^2}{|\mu _\ell - \mu _r|} \Big ), \end{aligned}$$

and

$$\begin{aligned} \frac{v_-}{\Vert v_- \Vert } = {1\atopwithdelims ()0} + o(1), \quad \frac{v_+}{\Vert v_+ \Vert } = {0\atopwithdelims ()1} + o(1). \end{aligned}$$

Hence the two first eigenfunctions of $\mathscr {L}$ are

$$\begin{aligned} \Psi _1 = e_\ell + o(1) = \Psi _\ell + o(1), \quad \Psi _2 = e_r+ o(1) = \Psi _r + o(1), \end{aligned}$$

and the result follows again.

### Application: a magnetic flea

Let us now turn to the effect indicated in the introduction and consider a slight perturbation of one of the wells in the originally symmetric pair—for definiteness we choose the right one—and inspect the effect on the eigenfunctions. For simplicity, we restrict to the situation when the perturbation is radially symmetric and at the same time supported away from the well center—our flea is thus weak but nonlocal; its support is an annular set—although *we conjecture that the effect will persist in the absence of the radial symmetry hypothesis*. We thus consider magnetic fields

$$\begin{aligned} B_\ell (x) = B^0(x-x_\ell ), \quad B_r(x) = B^0(x-x_r) + t_h \beta (x-x_r), \end{aligned}$$

where $t_h$ is a small *h*-dependent parameter and $B^0$, $\beta$ are radial functions satisfying the above assumptions.

#### Theorem 2.6

Assume that $\beta \geqslant 0$ is supported in $D(0,a) \setminus \lbrace 0 \rbrace$ and denote

$$\begin{aligned} R= \inf \lbrace |x|, x\in \textrm{supp} \, \beta \rbrace >0. \end{aligned}$$

Then for any $\varepsilon >0$ and *h* small enough we have

$$\begin{aligned} t_h\, e^{- (1+\varepsilon ) \frac{d_\ell (R)}{h}} \leqslant | \mu _\ell - \mu _r | \leqslant t_h\, e^{-(1-\varepsilon ) \frac{d_\ell (R)}{h}}. \end{aligned}$$

The flea-on-the-elephant effect then follows from the last theorem in combination with Corollary 2.4. We define the quantity

$$ I_0 = \frac{M}{2} - \frac{b_1L^2}{8} + \frac{b_1L}{2} \sqrt{\frac{L^2}{4}-\frac{M}{b_1}} - M \ln \left( 1 + \sqrt{ 1 - \frac{4\,M}{b_1 L^2}}\right) , $$

where *L* is the distance of the well centers, $b_1$ is the ‘background’ magnetic field intensity, and $M = \frac{1}{2\pi } \int _{\mathbb {R}^2} (2b_1 - B^0(x-x_\ell ) - B^0(x-x_r))\, \textrm{d}x$. Obviously, $I_0$ is the leading term of the quantity *I* from Theorem 2.3, $I = I_0 + \mathscr {O}(t_h)$.

#### Corollary 2.7

Let $L> (2+\sqrt{6})a$, then under the assumption of Theorem 2.6 we have the following dichotomy:

1. If $t_h \leqslant e^{- (1+\varepsilon )\frac{ 2 d_\ell (L/2) - d_\ell (R) + I_0}{h}}$ holds for some $\varepsilon >0$ independent of *h*, then the normalized eigenfunctions $\Psi _1$ and $\Psi _2$ of $\mathscr {L}$ are localized in both wells roughly equally,
  $$\begin{aligned} \int _{x_1 >0} |\Psi _j(x)|^2 \textrm{d}x = \frac{1}{2} + o(1),\quad j=1,2, \end{aligned}$$
  as $$h \rightarrow 0$$.
2. In contrast, if $h \gg t_h \geqslant e^{- (1-\varepsilon )\frac{ 2 d_\ell (L/2) - d_\ell (R) + I_0}{h}}$ holds for some $\varepsilon >0$ independent of *h*, then each of the normalized eigenfunctions $\Psi _1$ and $\Psi _2$ of $\mathscr {L}$ is localized dominantly in a single well,
  $$\begin{aligned} \int _{x_1>0} |\Psi _1(x) |^2 \textrm{d}x = o(1), \qquad \int _{x_1>0} |\Psi _2(x)|^2 \textrm{d}x = 1+ o(1), \end{aligned}$$
  as $$h \rightarrow 0$$.

In particular, since the right-hand side of the inequality in case (2) approaches zero faster than the left-hand one as $h \rightarrow 0$, even a very small perturbation of a symmetric double-well magnetic field can dramatically change the behavior of the eigenfunctions at the bottom of the spectrum. This result shows an approximate vanishing of tunneling by small asymmetry. It should be contrasted from [4], where an exact vanishing of tunneling is obtained even though the wells are still symmetric to each other.

#### Proof of Theorem 2.6

Since the perturbation is radially symmetric, by centering the single-well problems we see that $\mu _\ell =\mu _0$ and $\mu _r=\mu _t$ are the ground state energies of

$$\begin{aligned} \mathscr {M}_0 = (ih\nabla + A^0)^2, \quad \textrm{and} \quad \mathscr {M}_t = (ih \nabla + A^0 + t_h A^1)^2 \end{aligned}$$

respectively, where $A^0(x) = \int _{[0,x]} B^0(y)y^\perp \,\textrm{d}y$ and $A^1(x) = \int _{[0,x]} \beta (y) y^\perp \,\textrm{d}y$. Moreover, by Theorem 2.1 the ground state eigenfunctions are independent of angle; hence, $\mu _\ell$ and $\mu _r$ are the ground state energies of the following radial operators,

$$\begin{aligned} \mathscr {P}_0 = - \frac{h^2}{r} \partial _r r \partial _r + \frac{a(r)^2}{r^2}, \quad \mathscr {P}_t = - \frac{h^2}{r} \partial _r r \partial _r + \frac{(a(r) + t_h \alpha (r))^2}{r^2}, \end{aligned}$$

respectively, where $a(r):=\int _0^r B^0(s)s \textrm{d}s$ and $\alpha (r):= \int _0^r \beta (s)s\textrm{d}s$. We denote the associated normalized eigenfunctions by $\psi _0$ and $\psi _t$, i.e.,

$$\begin{aligned} \mathscr {P}_0 \psi _0 = \mu _\ell \psi _0, \quad \mathscr {P}_t \psi _t = \mu _r \psi _t. \end{aligned}$$

Note that the perturbation has exponentially small effect,

$$\begin{aligned} (\mathscr {P}_t - \mu _0) \psi _0 = \frac{2t_h \alpha (r)a(r) + t_h^2 \alpha (r)^2}{r^2} \psi _0 = \mathscr {O}(t_h\,e^{- \frac{d_\ell (R)}{h}}), \end{aligned}$$

since $\alpha$ is by assumption supported on $[R,\infty )$ and the function $\psi _0$ decays by Theorem 2.1 like $\exp (- d_\ell (r)/h)$; note that $d_r>d_\ell$. Using the min-max principle, we get $\inf \textrm{spec}(\mathscr {P}_t-\mu _0)\leqslant \langle \psi _0, (\mathscr {P}_t-\mu _0) \psi _0\rangle$ so that

$$\begin{aligned} | \mu _r - \mu _\ell | = \mathscr {O}\big (t_h\,e^{- \frac{d_\ell (R)}{h}}\big ) \end{aligned}$$

holds as $h\rightarrow 0$. Since the perturbation is bounded and the ground state eigenvalue of a single-well system is simple, we also have

$$\begin{aligned} \Vert \psi _t - \psi _0 \Vert = \mathscr {O}\big (t_h\,e^{- \frac{d_\ell (R)}{h}}\big ), \end{aligned}$$

which verifies the second inequality of Theorem 2.6.

To get a lower bound on the eigenvalue gap, we use a perturbation argument again. We write $\mathscr {P}_t = \mathscr {P}_0 + \mathscr {R}$, $\delta \mu = \mu _r - \mu _\ell$ and $\delta \psi = \psi _t - \psi _0$; then, the eigenvalue equation reads

$$\begin{aligned} ( \mathscr {P}_0 + \mathscr {R})(\psi _0 + \delta \psi ) = (\mu _0 + \delta \mu ) (\psi _0 + \delta \psi ), \end{aligned}$$

which implies

$$\begin{aligned} (\mathscr {P}_0 - \mu _0) \delta \psi + \mathscr {R} (\psi _0 + \delta \psi ) = \delta \mu (\psi _0 + \delta \psi ). \end{aligned}$$

Next we take the scalar product with $\psi _0$ to find that

$$\begin{aligned} \delta \mu ( 1 + \langle \delta \psi , \psi _0 \rangle ) = \langle \mathscr {R}(\psi _0 + \delta \psi ), \psi _0 \rangle . \end{aligned}$$

Furthermore, we have $\langle \delta \psi , \psi _0 \rangle = \mathscr {O}\big (e^{- \frac{d_\ell (R)}{h}}\big )$ and

$$\begin{aligned} | \langle \mathscr {R} \delta \psi , \psi _0 \rangle | \leqslant \Vert \delta \psi \Vert \Vert \mathscr {R} \psi _0 \Vert = \mathscr {O}(e^{- \frac{2d_\ell (R)}{h}}), \end{aligned}$$

where we have used Schwarz inequality together with the support properties of $\mathscr {R}$ and the decay of $\psi _0$ again. This yields

2.4 $$\begin{aligned} \delta \mu = \langle \mathscr {R} \psi _0, \psi _0 \rangle \big ( 1 + \mathscr {O}(e^{- \frac{d_\ell (R)}{h}}) \big ) +\mathscr {O}(e^{- \frac{2d_\ell (R)}{h}}). \end{aligned}$$

The leading term can be written explicitly as

$$\begin{aligned} \langle \mathscr {R} \psi _0, \psi _0 \rangle = \int _{R}^a \Big ( \frac{2t_h\alpha (r)a(r) + t_h^2\alpha (r)^2}{r^2} \Big ) |\psi _0(r)|^2 r \textrm{d}r > 0, \end{aligned}$$

which means that for any $\varepsilon >0$ we have the lower bound

$$\begin{aligned} \langle \mathscr {R} \psi _0, \psi _0 \rangle \geqslant \int _{R+\varepsilon }^a \Big ( \frac{2t_h\alpha (r)a(r) + t_h^2\alpha (r)^2}{r^2} \Big ) |\psi _0(r)|^2 r \textrm{d}r. \end{aligned}$$

Now we use the Laplace method, the exponential decay of $\psi _0$, and the fact that the integrated function is positive near $R+\varepsilon$, obtaining

$$\begin{aligned} \langle \mathscr {R} \psi _0, \psi _0 \rangle \geqslant C_\varepsilon t_h e^{-\frac{d_\ell (R+\varepsilon )(1 + \varepsilon )}{h}}, \end{aligned}$$

where the additional $(1+\varepsilon )$ was used to absorb the powers of $h^{-1}$ coming from the WKB approximation of Proposition 2.2. Using (2.4), we deduce that

$$\begin{aligned} |\delta \mu | \geqslant C_\varepsilon t_h e^{- \frac{d_\ell (R+\varepsilon )(1+\varepsilon )}{h}}, \end{aligned}$$

and the result follows since $\varepsilon$ was arbitrary.

#### Proof of Corollary 2.7

In view of Corollary 2.4, it is sufficient to compare the gap $|\mu _r - \mu _\ell |$ to the quantity *w* of Theorem 2.3; the $\varepsilon$ in the exponent can again absorb in the limit $h\rightarrow 0$ the power of *h* which *w* may contain.

## Proof of Theorem 2.3

Let us now return to the result on which all the previous discussion depends. The proof scheme is adapted from [9], where the case of mirror symmetric wells was considered.

### Construction of quasimodes

To change the gauge from *A* to $A_\ell$ or $A_r$, we introduce the functions $\sigma _\ell$ and $\sigma _r$ defined by

3.1 $$\begin{aligned} \sigma _\star (x) = \int _{[0,x]} (A-A_\star ) \cdot \textrm{d}s, \end{aligned}$$

where [0, *x*] is the segment connecting the point $x\in \mathbb {R}^2$ with the origin of coordinates and the dot means scalar product. Note that they satisfy

3.2 $$\begin{aligned} {\left\{ \begin{array}{ll} \nabla \sigma _\ell = A - A_\ell & \textrm{for} \quad x_1 < \frac{L}{2} - a,\\ \nabla \sigma _r = A-A_r & \textrm{for} \quad x_1 > - \frac{L}{2} + a. \end{array}\right. } \end{aligned}$$

In particular, for all $\Psi$ we have

3.3 $$\begin{aligned} \mathscr {L}( e^{i \frac{\sigma _\ell }{h}} \Psi ) = e^{i\frac{\sigma _\ell }{h}} \mathscr {L}_\ell \Psi , \quad \textrm{on} \quad x_1 < \frac{L}{2} - a, \end{aligned}$$

and a similar result holds also for $\mathscr {L}_r$.

Using rough decay estimates on the eigenfunctions, we easily find that the low-lying spectrum of the double-well operator $\mathscr {L}$ is situated close to $\lbrace \mu _{\ell ,1}, \mu _{r,1} \rbrace$.

#### Lemma 3.1

Assume that $\lambda \in \textrm{spec}( \mathscr {L})$ is such that for all *h* small enough the inequalities $|\lambda - \mu _{\ell ,1}| < |\lambda - \mu _{\ell ,2}|$ and $|\lambda - \mu _{r,1}| < |\lambda - \mu _{r,2}|$ hold. Then

$$\begin{aligned} \min \big ( |\lambda - \mu _{\ell ,1} |, |\lambda - \mu _{r,1}| \big ) = \mathscr {O}(h^\infty ). \end{aligned}$$

Note that the condition on $\lambda$ is meaningful when $\mu _{\ell ,1}$ and $\mu _{r,1}$ are close to each other (up to $o(h^2)$). This condition makes sense since our aim is to study cases when the right well is a perturbation of the left one. In particular, it is satisfied for $\lambda < \Lambda$; recall that $\Lambda$ is the minimum of the midpoints of the gaps between the two first eigenvalues left and right.

#### Proof

Let $\Psi$ be an eigenfunction of $\mathscr {L}$ with eigenvalue $\lambda$. We use two cutoff functions $\theta _\ell$ and $\theta _r$, smooth so that $\theta _\star \Psi$ belongs to the domain of $\mathscr {L}_\star$, and such that $\theta _\ell ^2 + \theta _r^2 =1$ and $\theta _\star$ equals one on a neighborhood of the well $D(x_\star , a)$. Using spectral theorem in combination with the fact that the closest eigenvalue of $\mathscr {L}_\star$ to $\lambda$ is $\mu _{\star ,1}$, we get the estimate

$$\begin{aligned} \Vert (\mathscr {L}_\ell - \lambda ) e^{-i \frac{\sigma _\ell }{h}} \theta _\ell \Psi \Vert ^2 + \Vert (\mathscr {L}_r - \lambda ) e^{-i \frac{\sigma _r}{h}} \theta _r \Psi \Vert ^2&\geqslant |\lambda - \mu _{\ell ,1}| \Vert \theta _\ell \Psi \Vert ^2 + |\lambda - \mu _{r,1}| \Vert \theta _r \Psi \Vert ^2 \\&\geqslant \min \big ( |\lambda - \mu _{\ell ,1}|, |\lambda - \mu _{r,1} | \big ) \Vert \Psi \Vert ^2. \end{aligned}$$

Using further relation (3.3) which holds on the support of $\theta _\ell$ and its right analogue, we arrive at

3.4 $$\begin{aligned} \Vert (\mathscr {L}-\lambda ) \theta _\ell \Psi \Vert ^2 + \Vert (\mathscr {L}-\lambda ) \theta _r \Psi \Vert ^2 \geqslant \min \big ( |\lambda - \mu _{\ell ,1}|, |\lambda - \mu _{r,1} | \big ) \Vert \Psi \Vert ^2. \end{aligned}$$

Since $\lambda$ is by assumption an eigenvalue of $\mathscr {L}$, the two expressions on the left-hand side can be bounded from above using the commutator,

$$\begin{aligned} \Vert (\mathscr {L} - \lambda ) \theta _\star \Psi \Vert ^2 = \Vert \big [ \mathscr {L}, \theta _\star \big ] \Psi \Vert ^2. \end{aligned}$$

The Agmon estimate (see, for instance, [13, Sect. 1.4]) tells us that $\Psi$ decays exponentially away from the wells, which is where the commutators are supported. Therefore,

$$\begin{aligned} \Vert (\mathscr {L} - \lambda ) \theta _\star \Psi \Vert ^2 = \mathscr {O}(h^\infty )\Vert \Psi \Vert ^2, \end{aligned}$$

which in combination with (3.4) yields the sought estimate.

In order to get better estimates on the eigenvalues and eigenfunctions, we construct next an appropriate eigenbasis starting from the left and right well ground states, $\Psi _\ell$ and $\Psi _r$, respectively. We use again smooth enough cutoff functions; this time they will be $\chi _\ell$, $\chi _r$ such that, for a sufficiently small $\eta >0$ we have

$$ \chi _\ell (x_1) = {\left\{ \begin{array}{ll} 1 & \mathrm{{if}} \quad x_1 < \frac{L}{2} - a - 2 \eta , \\ 0 & \mathrm{{if}} \quad x_1 > \frac{L}{2} - a-\eta , \end{array}\right. } $$

and $\chi _r(x_1) = \chi _\ell (-x_1)$. Then, we define

3.5 $$\begin{aligned} \psi _\ell = e^{i \frac{\sigma _\ell }{h}}\chi _\ell \Psi _\ell , \qquad \psi _r = e^{i \frac{\sigma _r}{h}} \chi _r \Psi _r, \end{aligned}$$

and furthermore, using the spectral projection $\Pi = \textbf{1}_{(-\infty ,\Lambda ]}\big ( \mathscr {L} \big )$, we put

$$\begin{aligned} g_\ell = \Pi \psi _\ell , \qquad g_r = \Pi \psi _r. \end{aligned}$$

Then we claim that $\psi _\ell$ and $\psi _r$ are exponentially good quasimodes for $\mathscr {L}$, and $g_\ell$, $g_r$ are small perturbations of them.

#### Lemma 3.2

For $\star \in \lbrace \ell , r \rbrace$, we have

$$\begin{aligned} \Vert (\mathscr {L}-\mu _\star ) \psi _\star \Vert = \mathscr {O}\left( e^{-d_\star \left( L - a - 3\eta \right) /h} \right) , \quad \Vert \psi _\star \Vert = 1 + \mathscr {O}\left( e^{-d_\star \left( L - a - 3\eta \right) /h} \right) . \end{aligned}$$

Moreover, if $|\mu _\ell - \mu _r| = o(h^2)$, we have

$$ \langle \mathscr {L} (g_\star - \psi _\star ), g_\star - \psi _\star \rangle = \mathscr {O}\left( e^{-2 d_\star \left( L - a - 4\eta \right) /h} \right) , \quad \Vert g_\star - \psi _\star \Vert = \mathscr {O}\left( e^{-d_\star \left( L - a - 4\eta \right) /h} \right) . $$

#### Proof

The proof follows the same lines as [9, Lemma 4.3]. Using (3.3) which holds on the support of $\chi _\ell$, we have

$$\begin{aligned} \Vert (\mathscr {L} - \mu _\ell ) \psi _\ell \Vert = \Vert (\mathscr {L}_\ell - \mu _\ell ) \chi _\ell \Psi _\ell \Vert = \Vert \big [ \mathscr {L}_\ell , \chi _\ell \big ] \Psi _\ell \Vert = \mathscr {O}\left( e^{-d_\ell \left( L - a - 3\eta \right) /h} \right) , \end{aligned}$$

where in the last estimate we used the exponential decay of $\Psi _\ell$ (Proposition 2.2) and the fact that the commutator is nonzero (and bounded) only where $\chi _\ell$ is non-constant. Taking into account that $\Psi _\ell$ is normalized, we get in a similar way the estimate on $\Vert \psi _\ell \Vert$, and the analogous inequalities hold for $\psi _r$.

The estimates on $g_\star - \psi _\star$ can be obtained in the following way: By definition of the spectral projection $\Pi$, we have

$$\begin{aligned} \langle (\mathscr {L}-\mu _\star ) (I-\Pi ) \psi _\star , (I-\Pi ) \psi _\star \rangle \geqslant (\Lambda - \mu _\star ) \Vert (I-\Pi ) \psi _\star \Vert ^2. \end{aligned}$$

Moreover, $\mathscr {L}$ commutes with its spectral projection $\Pi$, thus

$$\begin{aligned} \langle (\mathscr {L}-\mu _\star ) (I-\Pi ) \psi _\star , (I-\Pi ) \psi _\star \rangle \leqslant \Vert (\mathscr {L}-\mu _\star ) \psi _\star \Vert \Vert (I-\Pi ) \psi _\star \Vert . \end{aligned}$$

Combining the last two inequalities, we infer that

$$\begin{aligned} \Vert (I-\Pi ) \psi _\star \Vert \leqslant \big (\Lambda -\mu _\star \big )^{-1}\Vert (\mathscr {L}-\mu _\star ) \psi _\star \Vert . \end{aligned}$$

Since $\mu _\ell = \mu _r + o(h^2)$ and $\mu _{\ell ,2} - \mu _\ell \geqslant c h^2$ for some $c>0$ by Theorem 2.1, the gap between $\Lambda$ and $\mu _\star$ is of order $h^{2}$ which means that

$$\begin{aligned} \Vert (I-\Pi ) \psi _\star \Vert = \mathscr {O}\left( h^{-2} e^{-d_\star \left( L - a - 3\eta \right) /h} \right) . \end{aligned}$$

Furthermore, we can absorb the power factor in the exponential one changing $3\eta$ to $4 \eta$. Finally, to get the quadratic form bound, it is sufficient to use the Cauchy–Schwarz inequality,

$$\begin{aligned} \langle \mathscr {L} (I-\Pi ) \psi _\star , (I-\Pi ) \psi _\star \rangle \leqslant \Vert \mathscr {L} (I-\Pi ) \psi _\star \Vert \Vert (I-\Pi ) \psi _\star \Vert = \mathscr {O}\left( e^{-2 d_\star \left( L - a - 4\eta \right) /h} \right) , \end{aligned}$$

where the last estimate follows from $\Vert (\mathscr {L}-\mu _\star ) \psi _\star \Vert = \mathscr {O}\left( e^{-d_\star \left( L - a - 3\eta \right) /h} \right)$.

In fact, the pair $(g_\ell , g_r)$ spans the spectral subspace associated with $\Pi$.

#### Lemma 3.3

Assume that $\mu _\ell - \mu _r = o(h^2)$. Then for all *h* small enough, $(g_\ell ,g_r)$ is a basis of the subspace $\mathrm{{Ran}}\, \Pi$.

#### Proof

By Lemma 3.2, $(g_\ell , g_r)$ is a perturbation of $(\psi _\ell ,\psi _r)$, and therefore, $g_\ell$ and $g_r$ are linearly independent for *h* small enough. To prove that they form a basis of $\mathrm{{Ran}} \, \Pi$, it suffices to check that the only $\Psi \in \mathrm{{Ran}}\, \Pi$ orthogonal to both $g_\ell$ and $g_r$ is zero; note that by definition of vectors $g_\star$ this orthogonality requirement implies

3.6 $$\begin{aligned} \langle \Psi , \psi _\star \rangle = \langle \Pi \Psi , \psi _\star \rangle = \langle \Psi , g_\star \rangle = 0. \end{aligned}$$

We use the same cutoff functions $\theta _\ell$ and $\theta _r$ as in the proof of Lemma 3.1, that is, satisfying $\theta _\ell ^2 + \theta _r^2 =1$ and such that $\theta _\star$ equals one in a neighborhood of the well support $D(x_\star , a)$. Using the IMS localization formula [24, Prop. 4.2], we get

$$\begin{aligned} \langle \mathscr {L} \Psi , \Psi \rangle = \langle \mathscr {L} \theta _\ell \Psi , \theta _\ell \Psi \rangle + \langle \mathscr {L} \theta _r \Psi , \theta _r \Psi \rangle - h^2 \Vert (|\nabla \theta _\ell |^2 + |\nabla \theta _r|^2 ) \Psi \Vert ^2, \end{aligned}$$

and by Agmon estimates, $\Psi$ is exponentially small on the support of $\nabla \theta _\star$, so that

$$\begin{aligned} \langle \mathscr {L} \Psi , \Psi \rangle = \langle \mathscr {L} \theta _\ell \Psi , \theta _\ell \Psi \rangle + \langle \mathscr {L} \theta _r \Psi , \theta _r \Psi \rangle + \mathscr {O}(h^\infty )\Vert \Psi \Vert ^2. \end{aligned}$$

We insert the phase factor reflecting the gauge change and use (3.3) to obtain

$$\begin{aligned} \langle \mathscr {L} \Psi , \Psi \rangle = \langle \mathscr {L}_\ell e^{-i \frac{\sigma _\ell }{h}} \theta _\ell \Psi , e^{-i\frac{\sigma _\ell }{h}}\theta _\ell \Psi \rangle + \langle \mathscr {L}_r e^{-i \frac{\sigma _r}{h}} \theta _r \Psi , e^{-i \frac{\sigma _r}{h}}\theta _r \Psi \rangle + \mathscr {O}(h^\infty )\Vert \Psi \Vert ^2. \end{aligned}$$

Using next the spectral projections $\Pi _\star = |\Psi _\star \rangle \langle \Psi _\star |$, we find

$$\begin{aligned}  &   \langle \mathscr {L} \Psi , \Psi \rangle \geqslant \mu _\ell | \langle e^{-i \frac{\sigma _\ell }{h}} \theta _\ell \Psi , \Psi _\ell \rangle |^2 + \mu _r| \langle e^{-i \frac{\sigma _r}{h}} \theta _r \Psi , \Psi _r \rangle |^2 \\ +  &   \mu _{\ell ,2} \Vert (I-\Pi _\ell ) e^{-i \frac{\sigma _\ell }{h}} \theta _\ell \Psi \Vert ^2 + \mu _{r,2} \Vert (I-\Pi _r)e^{-i \frac{\sigma _r}{h}} \theta _r \Psi \Vert ^2+ \mathscr {O}(h^\infty )\Vert \Psi \Vert ^2. \end{aligned}$$

Note also that using the decay of $\psi _\star$, one is able to replace $\theta _\ell$ by the cutoff function $\chi _\ell$ of (3.5) up to exponentially small errors, so that

$$\begin{aligned} \langle e^{-i \frac{\sigma _\ell }{h}} \theta _\ell \Psi , \Psi _\ell \rangle = \langle \Psi , e^{i\frac{\sigma _\ell }{h}} \chi _\ell \Psi _\ell \rangle + \mathscr {O} (h^\infty ) \Vert \Psi \Vert = \mathscr {O}(h^\infty ), \end{aligned}$$

where in the last equality we have used the orthogonality assumption (3.6). In particular, $\Vert \Pi _\ell e^{-i \frac{\sigma _\ell }{h}} \theta _\ell \Psi \Vert = \mathscr {O}(h^\infty ) \Vert \Psi \Vert$, and we have

$$\begin{aligned} \langle \mathscr {L} \Psi , \Psi \rangle \geqslant \big (\min ( \mu _{\ell ,2}, \mu _{r,2} ) + \mathscr {O}(h^\infty ) \big ) \Vert \Psi \Vert ^2. \end{aligned}$$

However, vector $\Psi$ is by assumption in the range of $\Pi$, meaning that

$$\begin{aligned} \langle \mathscr {L} \Psi , \Psi \rangle \leqslant \min \big ( \frac{\mu _{\ell }+\mu _{\ell ,2}}{2}, \frac{\mu _r + \mu _{r,2}}{2} \big ) \Vert \Psi \Vert ^2. \end{aligned}$$

Since $\mu _\ell - \mu _r = o(h^2)$ by assumption and $\mu _{\star ,2}-\mu _{\star } \geqslant c h^2$ by Theorem 2.1, the last two inequalities are incompatible unless $\Psi =0$. Hence $\mathrm{{Ran}} \, \Pi \cap \lbrace g_\ell , g_r \rbrace ^\perp = \lbrace 0 \rbrace$, which means that $(g_\ell ,g_r)$ is a basis of the subspace $\mathrm{{Ran}}\, \Pi$.

### Interaction matrix

#### Proposition 3.4

Assume that $\mu _\ell - \mu _r = o(h^2)$. Then there exists an orthonormal basis $(e_\ell ,e_r)$ of $\mathrm{{Ran}} \, \Pi$ such that

$$\begin{aligned} e_\ell = \Psi _\ell + \mathscr {O}\big (h^\infty + |\langle \psi _r, \psi _\ell \rangle |\big ), \qquad e_r = \Psi _r + \mathscr {O}\big (h^\infty + |\langle \psi _r, \psi _\ell \rangle |\big ), \end{aligned}$$

and the restriction of $\mathscr {L}$ to $\mathrm{{Ran}} \, \Pi$ is in this basis represented by the matrix

$$ M = \begin{pmatrix} \mu _\ell &  w \\ \overline{w} &  \mu _r \end{pmatrix} + \mathscr {O}\big (|\langle \psi _\ell , \psi _r \rangle |^2 + |w|^2 + e^{- 2 d_\ell (L-a-4\eta )/h} + e^{-2d_r(L-a-4\eta )/h}\big ) $$

with $$w:= \langle \big ( \mathscr {L} - \frac{\mu _\ell + \mu _r}{2} \big ) \psi _\ell , \psi _r \rangle $$.

#### Proof

The action of $\mathscr {L}$ in the subspace $\mathrm{{Ran}} \, \Pi$ spanned by $(g_\ell ,g_r)$ is expressed by the matrix

$$ L = \begin{pmatrix} \langle \mathscr {L} g_\ell , g_\ell \rangle &  \langle \mathscr {L} g_\ell , g_r \rangle \\ \langle \mathscr {L} g_r, g_\ell \rangle &  \langle \mathscr {L} g_r, g_r \rangle \end{pmatrix}. $$

By virtue of Lemma 3.2, we can replace in this expression $g_\star$ by $\psi _\star$,

$$ L= \begin{pmatrix} \langle \mathscr {L} \psi _\ell , \psi _\ell \rangle &  \langle \mathscr {L} \psi _\ell , \psi _r \rangle \\ \langle \mathscr {L} \psi _r, \psi _{\ell } \rangle &  \langle \mathscr {L} \psi _r, \psi _r \rangle \end{pmatrix} + \mathscr {O} \big ( \mathcal E \big ) $$

with the error of order $\mathcal E = e^{- 2 d_\ell (L-a-4\eta )/h} + e^{-2d_r(L-a-4\eta )/h}$. By the same lemma, we can replace $\langle \mathscr {L} \psi _\star , \psi _\star \rangle$ by $\mu _\star$ with the same error, thus obtaining

$$ L = \begin{pmatrix} \mu _\ell &  w \\ \overline{w} &  \mu _r \end{pmatrix} + \frac{\mu _\ell + \mu _r}{2}T + \mathscr {O}\big ( \mathcal E \big ) $$

with $$w= \langle (\mathscr {L} - \frac{\mu _\ell + \mu _r}{2}) \psi _\ell , \psi _r \rangle $$ and

$$ T= \begin{pmatrix} 0 &  \langle \psi _\ell , \psi _r \rangle \\ \langle \psi _r, \psi _\ell \rangle &  0 \end{pmatrix}.$$

However, the basis $(g_\ell ,g_r)$ is not orthonormal. For this reason, we consider the Gram matrix

$$ G = \begin{pmatrix} \langle g_\ell , g_\ell \rangle &  \langle g_\ell , g_r \rangle \\ \langle g_r, g_\ell \rangle &  \langle g_r, g_r \rangle \end{pmatrix}, $$

the leading term of which can be by Lemma 3.2 expressed as

$$ G = I + T + \mathscr {O}(\mathcal E). $$

Then the basis ${e_\ell \atopwithdelims ()e_r} = G^{-\frac{1}{2}} {g_\ell \atopwithdelims ()g_r}$ is orthonormal, and the action of $\mathscr {L}$ in it is expressed by the matrix

$$ M = G^{-\frac{1}{2}} L G^{- \frac{1}{2}} = \begin{pmatrix} \mu _\ell &  w \\ \overline{w} &  \mu _r \end{pmatrix} +\mathscr {O}(|T|^2 + \mathcal {E} + |w|^2), $$

where we used Taylor expansion of the square root, $G^{-\frac{1}{2}}= I- \frac{1}{2} T + \mathscr {O}(T^2)$.

In order to prove Theorem 2.3, it remains to estimate *w* and the error terms $\langle \psi _\ell , \psi _r \rangle$ and $e^{-2d_\star (L-a-4\eta )/h}$. This is the purpose of the next section.

### Estimation of the interaction coefficient and the error terms

The sought estimates on *w*, $\langle \psi _\ell , \psi _r \rangle$ and of the error term $e^{-2d_\star (L-a-4\eta )/h}$ are given in Lemmata 3.5, 3.6 and 3.7, respectively. They are technical, and the two first ones are based on the Laplace method. We again follow the estimate ideas used in [9].

#### Lemma 3.5

There are a nonzero $c_0$ and $\nu \in \mathbb R$ such that

$$\begin{aligned} w = c_0 h^\nu e^{-S/h} \big (1+o(1)\big ) + \mathscr {O} \big ( (\mu _\ell - \mu _r) |\langle \psi _\ell , \psi _r \rangle | \big ) \end{aligned}$$

holds as $h \rightarrow 0$, where *S* is given by (2.3).

#### Proof

The proof is essentially the same as in [9, Section 5]; thus, we recall here only the main steps. In *w*, we replace $\mu _r$ by $\mu _\ell$ and we find

$$\begin{aligned} w = \tilde{w} + \mathscr {O}((\mu _\ell - \mu _r) \langle \psi _\ell , \psi _r \rangle ), \;\; \text {where}\;\; \tilde{w}:= \langle (\mathscr {L}-\mu _\ell ) \psi _\ell , \psi _r \rangle . \end{aligned}$$

Recalling the definition (3.5) of $\psi _\ell$ and using (3.3), we get

$$\begin{aligned} \tilde{w} = \langle [ \mathscr {L}, \chi _\ell ] e^{i \frac{\sigma _\ell }{h}} \Psi _\ell , e^{i \frac{\sigma _r}{h}} \Psi _r \rangle . \end{aligned}$$

Since $\mathscr {L}=P^2$ with $P=ih\nabla + A$, we can rewrite the commutator expression as

$$\begin{aligned} \tilde{w}&= \langle (P \cdot [P,\chi _\ell ] + [P,\chi _\ell ] \cdot P) e^{i \frac{\sigma _\ell }{h}}\Psi _\ell , e^{i \frac{\sigma _r}{h}} \Psi _r \rangle \\&= \langle [P_1, \chi _\ell ] e^{i \frac{\sigma _\ell }{h}}\Psi _\ell , P_1 e^{i \frac{\sigma _r}{h}} \Psi _r \rangle + \langle [P_1,\chi _\ell ] P_1 e^{i \frac{\sigma _\ell }{h}}\Psi _\ell , e^{i \frac{\sigma _r}{h}} \Psi _r \rangle \\&= ih \int _{x_1>0} \chi _\ell '(x_1) e^{\frac{i}{h} (\sigma _\ell - \sigma _r) }\big ( \Psi _\ell \overline{(ih\partial _1 + A_{r,1}) \Psi _r} + ((ih\partial _1 + A_{\ell ,1}) \Psi _\ell ) \overline{\Psi _r}\big ) \textrm{d}x. \end{aligned}$$

where we have used the fact that $\chi _\ell$ is independent of $x_2$ and $\chi _\ell '$ is supported in the half-plane $x_1>0$, together with $e^{-i \frac{\sigma _\star }{h}} P e^{i \frac{\sigma _\star }{h}} = P_\star$. Integrating then the obtained expression by parts, we get

$$\begin{aligned} \tilde{w}&= - ih\int _{\lbrace x:\,x_1 = 0 \rbrace } e^{\frac{i}{h}(\sigma _\ell - \sigma _r)} \big ( \Psi _\ell \overline{(ih\partial _1 + A_{r,1}) \Psi _r} + ((ih\partial _1 + A_{\ell ,1}) \Psi _\ell ) \overline{\Psi _r}\big ) \textrm{d}x_2\\&\quad + \int _{x_1>0} \chi _\ell (x_1) e^{\frac{i}{h}(\sigma _\ell - \sigma _r)} \big ( \Psi _\ell \overline{(ih\partial _1 + A_{r,1})^2 \Psi _r} - ((ih\partial _1 + A_{\ell ,1})^2 \Psi _\ell ) \overline{\Psi _r}\big ) \textrm{d}x\\&= -ih \int _{\{x:\,x_1=0\}} e^{\frac{i}{h}(\sigma _\ell - \sigma _r)} \big ( \Psi _\ell \overline{(ih\partial _1 + A_{r,1}) \Psi _r} + ((ih\partial _1 + A_{\ell ,1}) \Psi _\ell ) \overline{\Psi _r}\big ) \textrm{d}x_2 \\&\quad + \int _{x_1>0} \chi _\ell (x_1) e^{\frac{i}{h}(\sigma _\ell - \sigma _r)} \big ( \Psi _\ell \overline{\mathscr {L}_r \Psi _r} -\mathscr {L}_\ell \Psi _\ell \overline{\Psi _r}\big ) \textrm{d}x, \end{aligned}$$

where the last equality is justified using integration by parts with respect to $x_2$. Using further the eigenvalue equation for $\Psi _\ell$ and $\Psi _r$, we infer that

3.7 $$\begin{aligned}  &   w= -ih \int _{\{x:\,x_1=0\}} e^{\frac{i}{h}(\sigma _\ell - \sigma _r)} \big ( \Psi _\ell \overline{(ih\partial _1 + A_{r,1}) \Psi _r} + ((ih\partial _1 + A_{\ell ,1}) \Psi _\ell ) \overline{\Psi _r}\big ) \textrm{d}x_2 \nonumber \\  &   \quad + \mathscr {O}((\mu _r - \mu _\ell ) \langle \psi _\ell , \psi _r \rangle ). \end{aligned}$$

To estimate the above integral, we employ the Laplace method. In view of the complex phase involved, we have first to extend the function to complex values of the argument, namely

$$\begin{aligned} x_2 \mapsto x_2 + iq, \;\; \textrm{where} \;\; q = - \sqrt{ \frac{L^2}{4} - \frac{M_\ell + M_r}{b_1}} \end{aligned}$$

with $M_\star := \frac{1}{2\pi } \int _{\mathbb {R}^2} \big ( b_1 - B_\star \big ) \textrm{d}x.$ This can be done in view of the analyticity of $\Psi _\ell$ and $\Psi _r$ (see Lemma 4.2 and [9] for further details). We obtain

$$\begin{aligned} \mathcal I&:= -ih \int _{\{x:\,x_1=0\}} e^{\frac{i}{h}(\sigma _\ell - \sigma _r)} \big ( \Psi _\ell \overline{(ih\partial _1 + A_{r,1}) \Psi _r} + ((ih\partial _1 + A_{\ell ,1}) \Psi _\ell ) \overline{\Psi _r}\big ) \textrm{d}x_2 \\&= -ih \int _{\mathbb R} e^{\frac{i}{h}(\sigma _\ell - \sigma _r)(0,x_2+iq)} \big ( \Psi _\ell \overline{(ih\partial _1 + A_{r,1}) \Psi _r} \\&\quad + ((ih\partial _1 + A_{\ell ,1}) \Psi _\ell ) \overline{\Psi _r}\big )(0,x_2+iq) \textrm{d}x_2. \end{aligned}$$

Next we use Lemma 4.2 to replace $\Psi _\ell$ and $\Psi _r$ by the leading terms of their asymptotic expansions with the phase given by (5.1) and (5.4). This gives

$$\begin{aligned} \mathcal I = c_\ell c_r\, e^{- \frac{d_\ell (L/2) + d_r(L/2)}{h}} \int _{\mathbb R} e^{- \frac{g(x_2 + iq)}{h}} \omega (x_2 + i q) \,\textrm{d}x_2 \,\big ( 1+ o(1) \big ), \end{aligned}$$

with $$g(y) = \frac{b_1}{2} y^2 + i \frac{b_1 L}{2} y - (M_\ell + M_r) \ln \big ( 1 + i \frac{2 y}{L} \big ) $$ and

$$\begin{aligned} \omega (y)&= \big ( y+i \textstyle {\frac{L}{2}} \big ) \left( \frac{M_\ell + M_r}{ \frac{L^2}{4}+y^2} - b_1 \right) \left( \frac{\frac{b_1L^2}{8} - M_\ell }{\frac{b_1 L^2}{8} -M_\ell + \frac{b_1 y^2}{2}} \right) ^{\delta _\ell } \left( \frac{\frac{b_1L^2}{8} - M_r}{\frac{b_1L^2}{8} -M_r + \frac{b_1 y^2}{2}} \right) ^{\delta _r} \\&= g'(y) \left( \frac{\frac{b_1L^2}{8} - M_\ell }{\frac{b_1 L^2}{8} -M_\ell + \frac{b_1 y^2}{2}} \right) ^{\delta _\ell } \left( \frac{\frac{b_1L^2}{8} - M_r}{\frac{b_1L^2}{8} -M_r + \frac{b_1 y^2}{2}} \right) ^{\delta _r} \end{aligned}$$

with some $\delta _\star >0$. The function $x_2 \mapsto \textrm{Re}\, g(x_2+iq)$ has a unique minimum at $x_2=0$, and it is non-degenerate. For this reason, we are allowed to use the Laplace method by which there are $c_0 \ne 0$ and $\nu \in \mathbb R$ such that

$$\begin{aligned} \mathcal I = c_0\, h^\nu e^{- \frac{d_\ell (L/2) + d_r(L/2)+ g(iq)}{h}} (1+ o(1)) \end{aligned}$$

holds as $h\rightarrow 0$, and since

$$\begin{aligned}  &   g(iq) = \frac{M_\ell + M_r}{2} - \frac{b_1L^2}{8} + \frac{b_1L}{2} \sqrt{\frac{L^2}{4}-\frac{M_\ell + M_r}{b_1}} \\  &   \quad - (M_\ell + M_r) \ln \left( 1 + \sqrt{ 1 - \frac{4(M_\ell + M_r)}{b_1 L^2}}\right) , \end{aligned}$$

this concludes the proof.

#### Lemma 3.6

$$\langle \psi _\ell , \psi _r \rangle = \mathscr {O}(h^{-1}|w|)$$ holds as $$h \rightarrow 0$$.

#### Proof

By definition of $\psi _\ell$ and $\psi _r$, we have

$$\begin{aligned} \langle \psi _\ell , \psi _r \rangle = \int _{\mathbb {R}^2} \chi _\ell \chi _r\, e^{\frac{i}{h}(\sigma _\ell - \sigma _r)} \Psi _\ell \overline{\Psi }_r \,\textrm{d}x. \end{aligned}$$

This integral can be estimated in a way similar to that of Lemma 3.5. We recall that $\chi _\ell$ and $\chi _r$ depend on $x_1$ only. On the support of $\chi _\ell \chi _r$, the functions $\Psi _\ell$ and $\Psi _r$ are analytic with respect to $x_2$. This makes it possible to extend them to complex values of the argument, $x_2 \mapsto x_2 +iq(x_1)$, where

$$\begin{aligned} q(x_1) = - \sqrt{\left( \frac{L}{2} - |x_1| \right) ^2 - \frac{M_\ell + M_r}{b_1}} \end{aligned}$$

so that $x_2$ stays in the analyticity domain of $\Psi _\ell$ and $\Psi _r$ and we get

$$\begin{aligned} \langle \psi _\ell , \psi _r \rangle = \int _{\mathbb {R}^2} \left( \chi _\ell \chi _r\, e^{\frac{i}{h}(\sigma _\ell - \sigma _r)} \Psi _\ell \overline{\Psi }_r \right) (x_1, x_2+iq(x_1)) \,\textrm{d}x. \end{aligned}$$

Now we use the asymptotic formula from Lemma 4.2 which yields

$$\begin{aligned} \langle \psi _\ell , \psi _r \rangle = c_\ell c_r h^{-1} e^{- \frac{d_\ell (L/2) + d_r(L/2)}{h} } \int _{\mathbb {R}^2} e^{- \frac{f(x_1,x_2+iq_1)}{h}} \tilde{\omega }(x_1,x_2+iq(x_1)) \,\textrm{d}x, \end{aligned}$$

with

3.8 $$\begin{aligned}  &   f(x) = \frac{b_1}{4} \left( |x-x_\ell |^2 + |x-x_r|^2 - \frac{L^2}{2} +2iLx_2 \right) \nonumber \\  &   \quad - M_\ell \ln \left( \frac{2}{\,}L \left( \frac{L}{2} +x_1 +ix_2 \right) \right) - M_r \ln \left( \frac{2}{\,}L \left( \frac{L}{2} -x_1 +ix_2 \right) \right) \end{aligned}$$

and

3.9 $$\begin{aligned} \tilde{\omega }(x_1,x_2) = \left( \frac{\frac{b_1L^2}{8} - M_\ell }{\frac{b_1}{2}\left( \frac{L}{2} + x_1 \right) ^2 +\frac{b_1x_2^2}{2} -M_\ell } \right) ^{\delta _\ell } \left( \frac{\frac{b_1L^2}{8} - M_r}{\frac{b_1}{2}\left( \frac{L}{2} - x_1 \right) ^2 +\frac{b_1x_2^2}{2} -M_r} \right) ^{\delta _r}. \end{aligned}$$

The function $x_1 \mapsto \textrm{Re} f(x_1,x_2+iq(x_1))$ has a non-degenerate global minimum at $x_1=0$, and consequently, the Laplace method gives

3.10 $$\begin{aligned} | \langle \psi _\ell , \psi _r \rangle | \leqslant C h^{- \frac{1}{2}} e^{- \frac{d_\ell (L/2) + d_r(L/2)}{h} } \int _{\mathbb {R}} e^{- \mathrm{{Re}} \frac{f(0,x_2+iq(0))}{h}} \tilde{\omega }(0,x_2+iq(0)) \,\textrm{d}x_2. \end{aligned}$$

Note further that $f(0,x_2) = g(x_2)$ and $\tilde{\omega }(x_2) = \frac{1}{g'(x_2)}\omega (x_2)$ holds with the notations of the proof of Lemma 3.5, thus we get

3.11 $$\begin{aligned} | \langle \psi _\ell , \psi _r \rangle | \leqslant C h^{-1}|w|, \end{aligned}$$

which concludes the proof.

Finally, we need an estimate from which the condition on well spacing *L* follows:

#### Lemma 3.7

If $L > (2+\sqrt{6}) a$, then for $\star \in \lbrace \ell , r \rbrace$ we have

$$\begin{aligned} 2d_\star (L-a) > S. \end{aligned}$$

In particular, if $\eta >0$ is small enough, then $e^{-2d_\star (L-a-4\eta )/h} = o(h^\nu e^{-S/h})$ holds for all $\nu \in \mathbb R$.

#### Proof

The proof is identical with that of [9, Lemma 5.2].

Putting the above results together, we complete the proof of Theorem 2.3.

## Data Availability

Data sharing not applicable to this article as no datasets were generated or analyzed during the current study.

## About the single-well ground state

### Lemma 4.1

In the exterior of the well, $|x-x_\star | \geqslant a$, we have

$$\begin{aligned} \Psi _\star (x) = C_\star |x-x_\star |^{\gamma _{\star }} \int _0^{\infty } e^{- \frac{b_1}{4\,h} (1+2t) |x-x_\star |^2} m_\star (t) \,\textrm{d}t \end{aligned}$$

with

$$\begin{aligned} \gamma _\star = \frac{M_\star }{h}, \quad \delta _\star =\frac{b_1 h - \mu _\star }{2hb_1}, \quad m_\star (t) = t^{\delta _\star -1} (1+t)^{\gamma _\star - \delta _\star }, \end{aligned}$$

where $$M_\star = \frac{1}{2\pi } \int _{\mathbb {R}^2} \big ( b_1 - B_\star (x) \big ) \textrm{d}x$$ and

$$\begin{aligned} C_\star = \frac{\Psi _\star (0)}{|x_\star |^{\gamma _\star }} \Big ( \int _0^\infty e^{- \frac{b_1}{4\,h}(1+2t)|x_\star |^2} m_\star (t) \textrm{d}t \Big )^{-1}. \end{aligned}$$

### Proof

The claim is established using the Kummer functions, see [9].

### Lemma 4.2

For $x_1 \in \big (- \frac{L}{2} +a, \frac{L}{2} -a \big )$, the function $x_2 \mapsto \Psi _\star (x_1,x_2)$ has a holomorphic extension in the second argument to the region

$$\begin{aligned} \Omega (x_1) = \big \lbrace x_2 \in \mathbb C, \, \big (x_1 \pm \frac{L}{2}\big )^2 + (\mathrm{{Re}} x_2)^2 - (\mathrm{{Im}} x_2)^2 > a^2 \big \rbrace . \end{aligned}$$

Moreover, there exists a positive constant $c_\star >0$ such that

$$\begin{aligned} \Psi _\star (x) = c_\star h^{- \frac{1}{2}} \left( \frac{\frac{b_1L^2}{8} - M_\star }{\frac{b_1}{2}|x-x_\star |^2 -M_\star } \right) ^\delta \frac{|x-x_\star |^{\gamma _\star }}{|x_\star |^{\gamma _\star }} e^{- \frac{d_\star (L/2)}{h}} e^{- \frac{b_1}{4\,h}(|x-x_\star |^2 - |x_\star |^2)} \big ( 1+ \mathscr {O}(h) \big ) \end{aligned}$$

holds locally uniformly for $x_1 \in \big (- \frac{L}{2} +a, \frac{L}{2} -a \big )$ and $x_2 \in \Omega (x_1)$.

### Proof

The claim follows from Lemma 4.1. Since $M_\star < \frac{b_1 a^2}{2}$, the integral defining $\Psi _\star$ is holomorphic on $\Omega (x_1)$. One can estimate $\Psi _\star$ and $C_\star$ using the Laplace method; the value of $\Psi _\star (0)$ is given by the WKB approximation, Proposition 2.2.

## About the complex phase

In the above reasoning, we needed the phase shift $\sigma _r - \sigma _\ell$ defined by (3.1) expressing the gauge difference between the two wells. It can be calculated as follows:

$$\begin{aligned} \sigma _r(x) - \sigma _\ell (x)&= \int _{[0,x]} (A_\ell - A_r) \cdot \textrm{d}s \\&=\int _0^{x_1} (A_\ell - A_r)_{1}(u,0) \,\textrm{d}u + \int _0^{x_2} (A_\ell - A_r)_2 (x_1,u) \,\textrm{d}u \\&= \int _0^{x_2} (A_\ell - A_r)_2(x_1,u) \,\textrm{d}u, \end{aligned}$$

with

$$\begin{aligned} A_\star (x) = \int _0^1 B_\star (x_\star + t(x-x_\star ))t \,\textrm{d}t \begin{pmatrix} -x_2 \\ x_1 \pm \frac{L}{2} \end{pmatrix} = \left( \frac{b_1}{2} - \frac{M_\star }{|x-x_\star |^2} \right) \begin{pmatrix} -x_2 \\ x_1 \pm \frac{L}{2} \end{pmatrix} \end{aligned}$$

and the sign + corresponding $A_\ell$ and −, respectively, to $A_r$. In particular, we have

$$\begin{aligned} A_{\star ,2}(x) = \frac{b_1}{2} \big (x_1 \pm \frac{L}{2}\big ) - \frac{M_\star (x_1 \pm \frac{L}{2})}{(x_1 \pm \frac{L}{2})^2 + x_2^2} \end{aligned}$$

and

$$\begin{aligned} A_{\ell ,2}(x) - A_{r,2}(x) = \frac{b_1 L}{2} +\frac{M_r (x_1 - \frac{L}{2})}{(x_1 - \frac{L}{2})^2 + x_2^2} - \frac{M_\ell (x_1 + \frac{L}{2})}{(x_1 + \frac{L}{2})^2 + x_2^2}. \end{aligned}$$

Consequently, the phase difference equals

5.1 $$\begin{aligned} \sigma _r(x) - \sigma _\ell (x) = \frac{b_1 L}{2}x_2 - M_r \arctan \left( \frac{x_2}{\frac{L}{2} - x_1} \right) - M_\ell \arctan \left( \frac{x_2}{x_1 + \frac{L}{2}} \right) . \end{aligned}$$

In Lemma 3.6, we have also encounter the phase

5.2 $$\begin{aligned}  &   f(x) {=} \frac{b_1}{4} \left( |x-x_\ell |^2 {+} |x-x_r|^2 {-} \frac{L^2}{2} \right) {-} M_r \ln \left( \frac{|x-x_r|}{|x_r|} \right) {-} M_\ell \ln \left( \frac{|x-x_\ell |}{|x_\ell |} \right) \nonumber \\  &   + i \frac{b_1 L}{2} x_2 - i M_r \arctan \left( \frac{x_2}{\frac{L}{2} - x_1} \right) -i M_\ell \arctan \left( \frac{x_2}{x_1 + \frac{L}{2}} \right) . \end{aligned}$$

which can be rewritten using the complex logarithm

5.3 $$\begin{aligned}  &   f(x) = \frac{b_1}{4} \left( |x-x_\ell |^2 + |x-x_r|^2 - \frac{L^2}{2} +2iLx_2 \right) \nonumber \\  &   \quad - M_\ell \ln \left( \frac{2}{\,}L \left( \frac{L}{2} +x_1 +ix_2 \right) \right) - M_r \ln \left( \frac{2}{\,}L \left( \frac{L}{2} -x_1 +ix_2 \right) \right) . \end{aligned}$$

Note that the function $g(y) = f(0,y)$ of Lemma 3.5 is given by

5.4 $$\begin{aligned} g(y) = \frac{b_1y^2}{4} + i \frac{b_1 Ly }{2} - (M_\ell + M_r) \ln \left( 1 + i \frac{2y}{L} \right) . \end{aligned}$$

## References

[1] Alfa, K.A.: Tunneling effect in two dimensions with vanishing magnetic fields. J. Spectr. Theory **14**(3), 837–889 (2024)

[2] Bonnaillie-Noël, V., Hérau, F., Raymond, N.: Purely magnetic tunneling effect in two dimensions. Invent. Math. **227**(2), 745–793 (2022)

[3] Fefferman, C., Shapiro, J., Weinstein, M.I.: Lower bound on quantum tunneling for strong magnetic fields. SIAM J. Math. Anal. **54**(1), 1105–1130 (2022)

[4] Fefferman, C.L., Shapiro, J., Weinstein, M.I.: Magnetic double-wells: absence of tunneling (2025). arXiv:2509.02857

[5] Fefferman, C.L., Shapiro, J., Weinstein, M.I.: Magnetic double-wells: lower bounds on tunneling (2025). arXiv:2511.13470

[6] Fournais, S., Guedes Bonthonneau, Y., Morin, L., Raymond, N.: Tunneling between magnetic wells in two dimensions (2025). arXiv:2502.17290

[7] Fournais, S., Helffer, B.: Spectral Methods in Surface Superconductivity. Progress in Nonlinear Differential Equations and their Applications, vol. 77. Birkhäuser Boston Inc, Boston (2010)

[8] Fournais, S., Helffer, B., Kachmar, A.: Tunneling effect induced by a curved magnetic edge. In: The Physics and Mathematics of Elliott Lieb—The 90th Anniversary, vol. I, pp. 315–350. EMS Press, Berlin (2022)

[9] Fournais, S., Morin, L., Raymond, N.: Purely magnetic tunneling between radial magnetic wells. Rev. Mat. Iberoam (2025)

[10] Graffi, S., Grecchi, V., Jona-Lasinio, G.: Tunnelling instability via perturbation theory. J. Phys. A: Math. Gen. **17**, 2935–2944 (1984)

[11] Guedes Bonthonneau, Y., Nguyen Duc, T., Raymond, N., Vũ Ngọc, S.: Magnetic WKB constructions on surfaces. Rev. Math. Phys. **33**(7), 2150022 (2021)

[12] Guedes Bonthonneau, Y., Raymond, N.: WKB constructions in bidimensional magnetic wells. Math. Res. Lett. **27**(3), 647–663 (2020)

[13] Guedes Bonthonneau, Y., Raymond, N., Vũ Ngọc, S.: Exponential localization in 2D pure magnetic wells. Ark. Mat. **59**(1), 53–85 (2021)

[14] Helffer, B.: Semi-classical Analysis for the Schrödinger Operator and Applications. Lecture Notes in Mathematics, vol. 1336. Springer, Berlin (1988)

[15] Helffer, B., Kachmar, A.: Quantum tunneling in deep potential wells and strong magnetic field revisited. Pure Appl. Anal. **6**(2), 319–352 (2024)

[16] Helffer, B., Kachmar, A., Sundqvist, M.P.: Flux and symmetry effects on quantum tunneling. Mathematische Annalen (2024)

[17] Helffer, B., Kordyukov, Y.: Accurate semiclassical spectral asymptotics for a two-dimensional magnetic Schrödinger operator. Ann. Henri Poincaré **16**(7), 1651–1688 (2015)

[18] Helffer, B., Mohamed, A.: Semiclassical analysis for the ground state energy of a Schrödinger operator with magnetic wells. J. Funct. Anal. **138**(1), 40–81 (1996)

[19] Helffer, B., Sjöstrand, J.: Multiple wells in the semi-classical limit I. Commun. Part. Differ. Equ. **9**, 337–408 (1984)

[20] Helffer, B., Sjöstrand, J.: Effet tunnel pour l’équation de schrödinger avec champ magnétique. Annali della Scuola Superiore di Pisa **14**(4), 625–657 (1987)

[21] Himpsel, F.J.: Magnetic quantum wells. J. Phys.: Condens. Matter **11**, 9483–9494 (1999)

[22] Jona-Lasinio, G., Martinelli, F., Scoppola, E.: New approach to the semiclassical mechanics I. multiple tunnelings in one dimension. Commun. Math. Phys. **80**, 2223–254 (1981)

[23] Morin, L.: Tunneling effect between radial electric wells in homogeneous magnetic field. Lett. Math. Phys. **114**, 29 (2024)

[24] Raymond, N.: Bound States of the Magnetic Schrödinger Operator, volume 27 of EMS Tracts in Mathematics. EMS Publication (2017)

[25] Raymond, N., Vũ Ngọc, S.: Geometry and spectrum in 2D magnetic wells. Ann. Inst. Fourier (Grenoble) **65**(1), 137–169 (2015)

[26] Reijniers, J., Peeters, F.M., Matulis, A.: Quantum states in a magnetic antidot. Phys. Rev. B **59**, 2816–2823 (1999)

[27] Simon, B.: Semiclassical analysis of low lying eigenvalues IV. The flea on the elephant. J. Funct. Anal. **63**, 123–136 (1985)

